# A rare case of arteriovenous fistula formation in a patient with inferior vena cava thrombosis, successfully managed with endovascular recanalisation

**DOI:** 10.1259/bjrcr.20190007

**Published:** 2019-11-15

**Authors:** Vanya Joshi, Frances Sheehan, Alexander Chapman

**Affiliations:** 1 St Peter’s Hospital, Chertsey, United Kingdom

## Abstract

Inferior vena cava (IVC) filters are recommended for patients with proximal deep vein thrombosis (DVT) who are not eligible for anticoagulation. Long-dwelling filters are well-known to be associated with the development of IVC thrombosis. Chronic caval occlusion can lead to a severe post-thrombotic syndrome (PTS), with manifestations of chronic venous insufficiency in the lower extremities. Animal studies have shown that post-thrombotic inflammation can trigger the development of an arteriovenous fistula (AVF), however, there is limited evidence for this phenomenon in patients with PTS. We describe the case of a spontaneous AVF in a patient with long-standing IVC thrombosis. It was postulated that the AVF could be compounding the venous hypertension and severe swelling of his lower extremities. The case additionally demonstrates the successful results of endovascular recanalisation for an occluded filter in the presence of an AVF.

## Case presentation

A 64-year-old male presented with right-sided weakness and CT head confirmed a haemorrhagic stroke in the left basal ganglia. On admission, he was recruited into the CLOTS trial, a multicenter randomised control trial assessing the use of intermittent pneumatic compression (IPC) in immobile stroke patients.^1^ A baseline ultrasound (US) Doppler scan of his lower extremities on day 2 post-stroke showed no thrombus and IPC was commenced.

A follow-up US Doppler scan was conducted on day 10 post-stroke, as per trial protocol. Although the patient was asymptomatic, the US showed non-occlusive thrombi within the right common femoral and superficial femoral veins, with occlusive thrombi in the right popliteal and calf veins. There was no evidence of thrombus in the left leg. Anticoagulation and thrombolysis were contraindicated due to the recent haemorrhagic stroke, so a decision was made to insert a Günther Tulip^®^ Vena Cava filter (Cook Medical, IA). The procedure was performed the following day, via an uncomplicated right femoral approach.

A repeat US Doppler on day 30 post-stroke showed extension of the thrombus into the iliac veins bilaterally, and into the IVC. Long-term pharmacological anticoagulation was initiated, but the IVC filter was left *in situ* due to the extent of the thrombosis.

**Figure 1.**
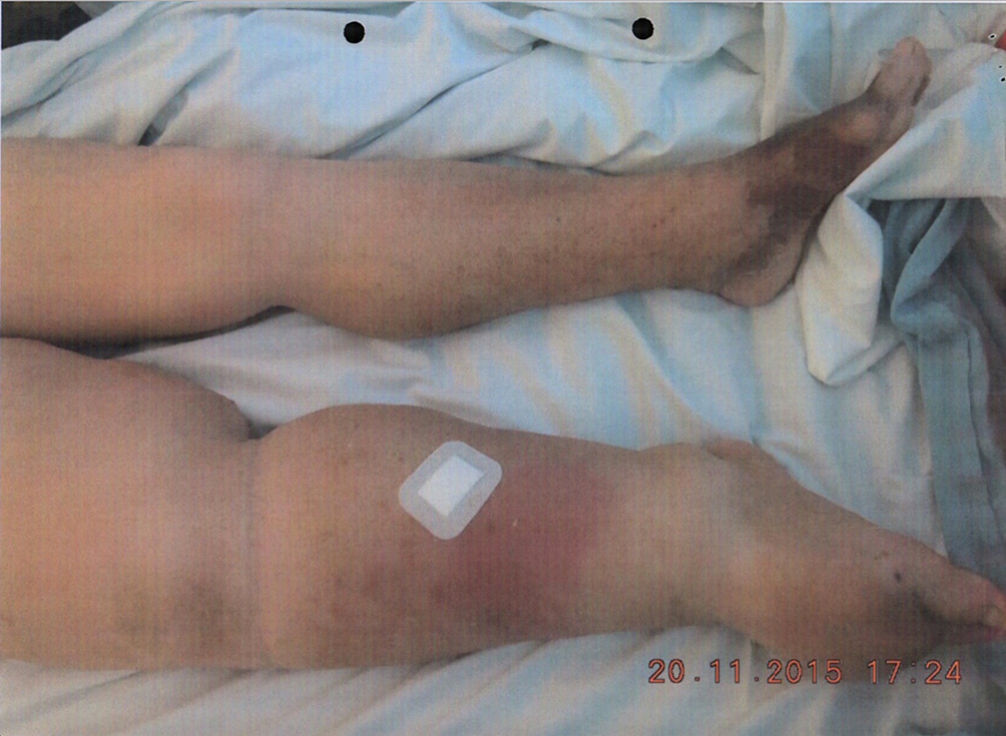
There is extensive oedema extending from the right lower calf up to the groin. There are signs of active ulceration, venous eczema and pigmentation changes. The left leg shows moderate signs of venous disease, however the swelling is isolated to the right-hand side.

Over the following year, the patient was lost to follow up. He re-presented 18 months later with features of PTS - including extensive swelling and oedema of his right leg extending up to his groin, pigmentation changes, venous eczema and active ulceration affecting his right lower calf and lateral thigh (Figure 1). His left calf demonstrated pigmentation changes, but no swelling. He scored 20/30 points using the Revised Venous Clinical Severity Score.^2^ Examination further revealed prominent dilated tortuous superficial abdominal veins. The swelling of his right leg caused him excruciating pain, which compounded his residual mobility issues post-stroke, and rendered him wheelchair bound.

## Investigations/IMAGING FINDINGS

**Figure 2.**
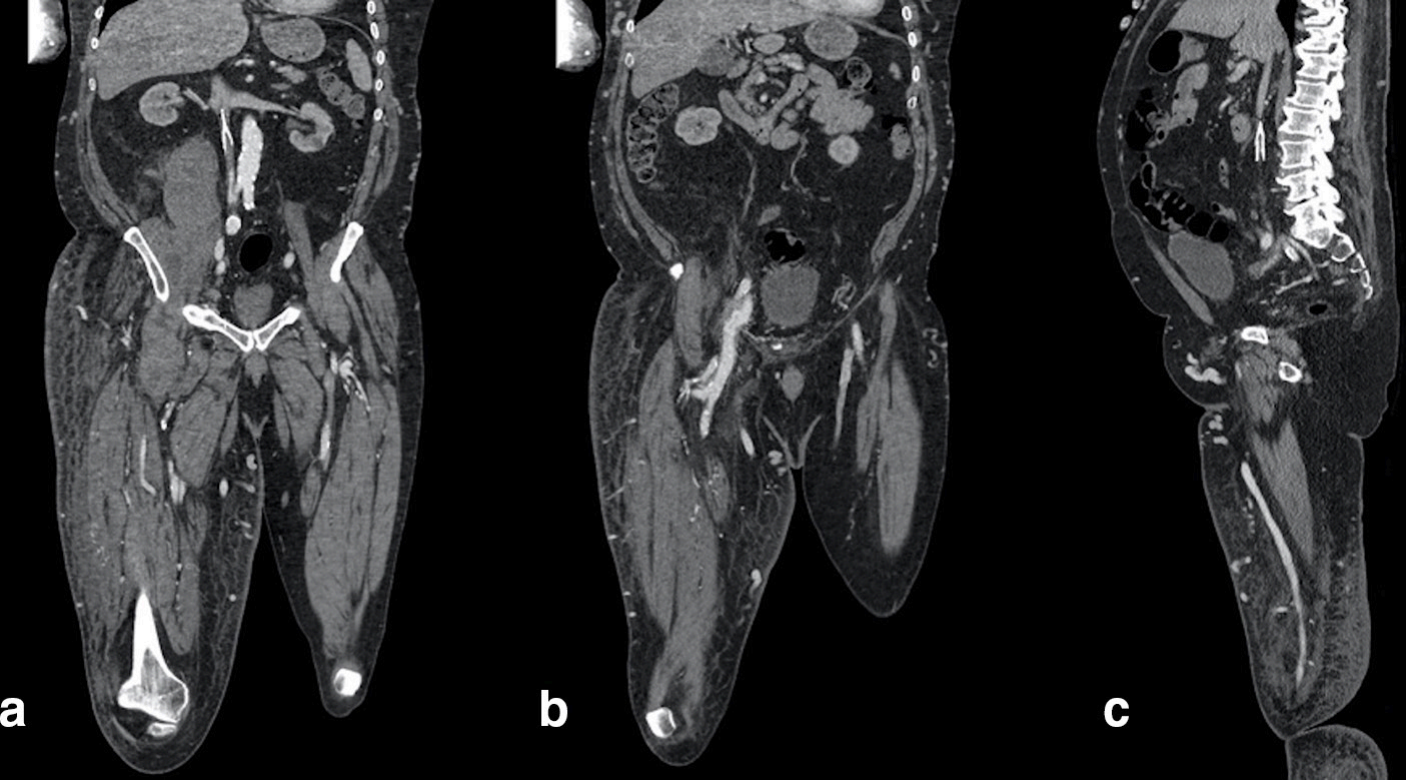
(a) CT angiogram (coronal) demonstrates severe post-thrombotic occlusion of the infrarenal IVC, with a collapsed caval filter *in situ*. (b) The right common femoral vein appears dilated and abnormally enhanced with surrounding inflammatory change, in keeping with the presence of an AVF. The vein drains principally via numerous varicose dilated superficial veins overlying the anterior abdominal wall. (c) CT angiogram (saggital) demonstrates the collapsed filter embedded in the thrombosed IVC. There is gross oedema of the right leg consistent with impaired venous drainage.

A CT angiogram (Figure 2a, b and c) demonstrated chronic occlusion of the IVC below the filter and extensive superficial abdominal collaterals. In addition, there were abnormal soft-tissue inflammatory changes surrounding the right common femoral and external iliac veins, with early enhancement of the right common femoral vein (Figure 2b). This suggested the possibility of the presence of an AVF.

**Figure 3.**
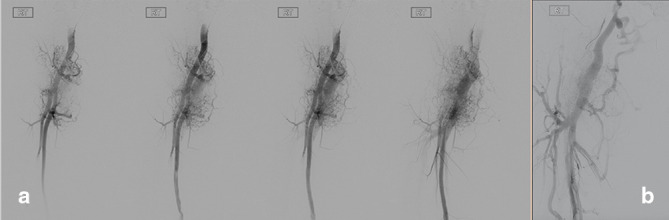
(a) Right-sided lower limb catheter angiogram demonstrates a complex vascular malformation arising from the distal external iliac artery, common femoral artery, profunda femoris and superficial femoral artery. (b) There is early venous filling of the common femoral vein, external iliac vein and extensive collaterals, in keeping with an AVF.

Catheter angiography (Figure 3a and b) confirmed the presence of an AVF with immediate filling of the femoral and iliac veins following the arterial injection. There was considerable neovascularisation surrounding the lesion, in keeping with a chronic AVF.

## Treatment

A vascular surgical opinion postulated that the AVF, in the presence of occlusive thrombus, was contributing to venous hypertension, and thus the swelling of his right limb. An open surgical repair of the AVF was attempted, however, an intraoperative decision was made to abandon the surgery due to the complexity of the vasculature.

Stocking compression and massage therapy were trialed, but his symptoms did not improve. The leg swelling was physically disabling and causing the patient to feel depressed, and he was considering a below-knee amputation. However, he was deemed a poor candidate for this due to the significant oedema that would impair wound healing.

**Figure 4.**
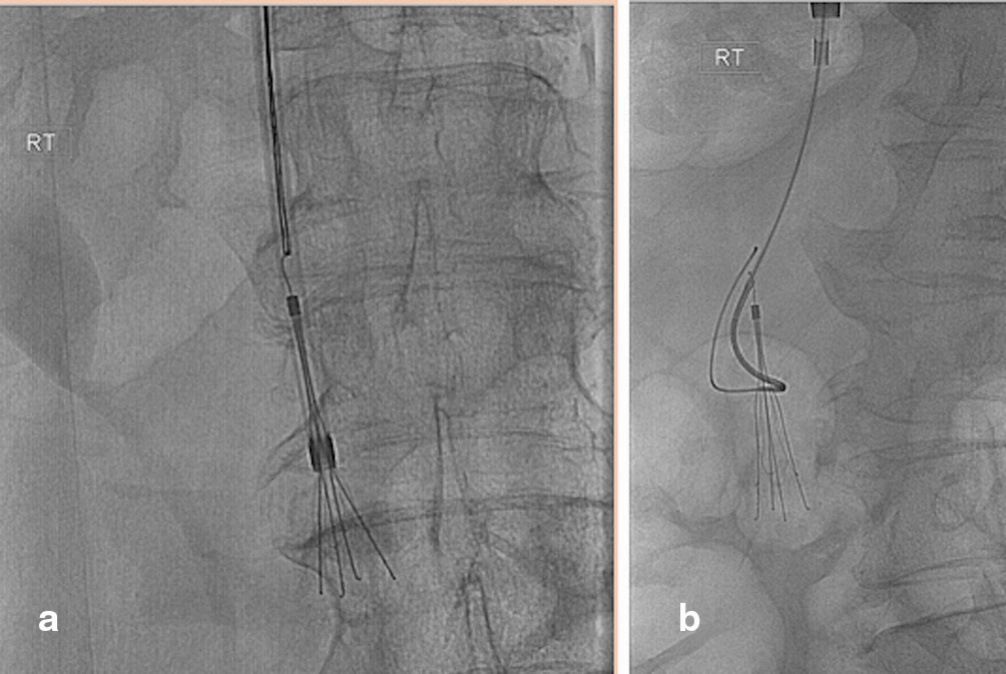
(a) In an attempt to retrieve the filter, the superior filter hook has been snared, however maximal traction has simply straightened the hook. The IVCF remains firmly adherent to the IVC wall. (b) An alternative approach to retrieval has been attempted by hooking the legs of the filter, however this was also unsuccessful.

Following multi disciplinary team (MDT) involvement and discussion with other centres, it was decided to attempt to remove the IVC filter. A 10Fr CloverSnare^™^ 4-Loop Vascular Retriever (Cook Medical, IA) was initially deployed using a triaxial sytem with both 16F and 20F Gore^®^ DrySeal Sheaths (Gore, Az). The filter hook was snared however, prolonged forceful traction resulted in straightening of the superior hook without successful retrieval (Figure 4a). A further unsuccessful attempt was made with a through and through ZIPwire^™^ hydrophilic glide wire (Boston Scientific, MA) passing between the legs and body of the filter (Figure 4b). A final attempt was made to engage the proximal hook with EndoJaw^™^ disposable bronchoscopic biopsy forceps (Olympus Medical Systems Corp, Tokyo, Japan), but it was similarly not possible to remove the filter despite maximal traction. As a result of these extensive attempts, the removal was abandoned and the filter was left *in situ*.

**Figure 5.**
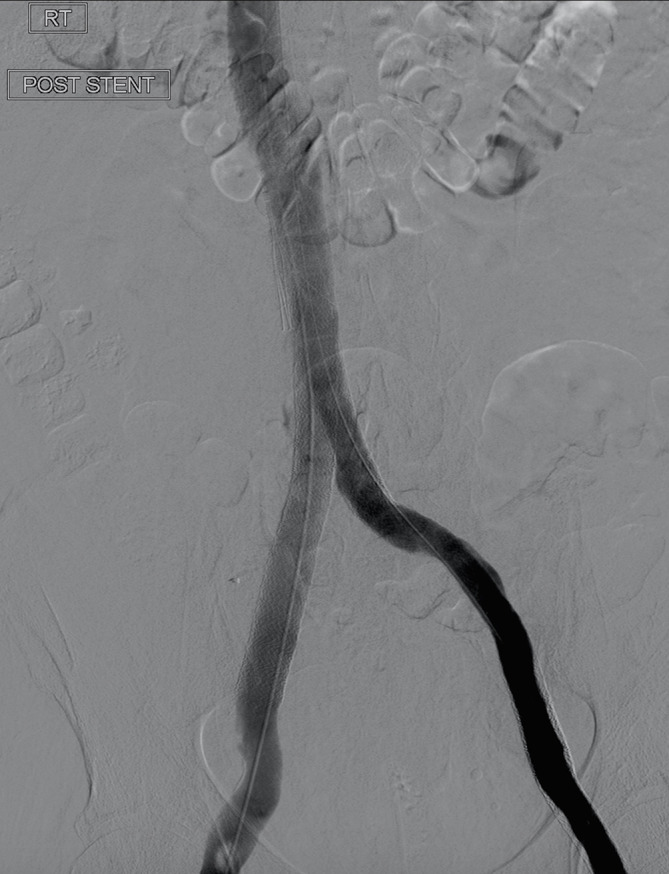
Following retrograde recanalisation of the left iliac system and IVC, a good venographic result has been achieved, with swift flow through both stents, particularly on the right side-in view of the pre-existing AVF.

It was finally determined that endovascular recanalisation of the IVC would be the best approach. Vici venous stents^®^ (Boston Scientific, MA) were inserted via the right external iliac vein and left common femoral vein to the level of the renal vein confluence, with post-deployment venoplasty (Figure 5).

## Outcome and follow-up

**Figure 6.**
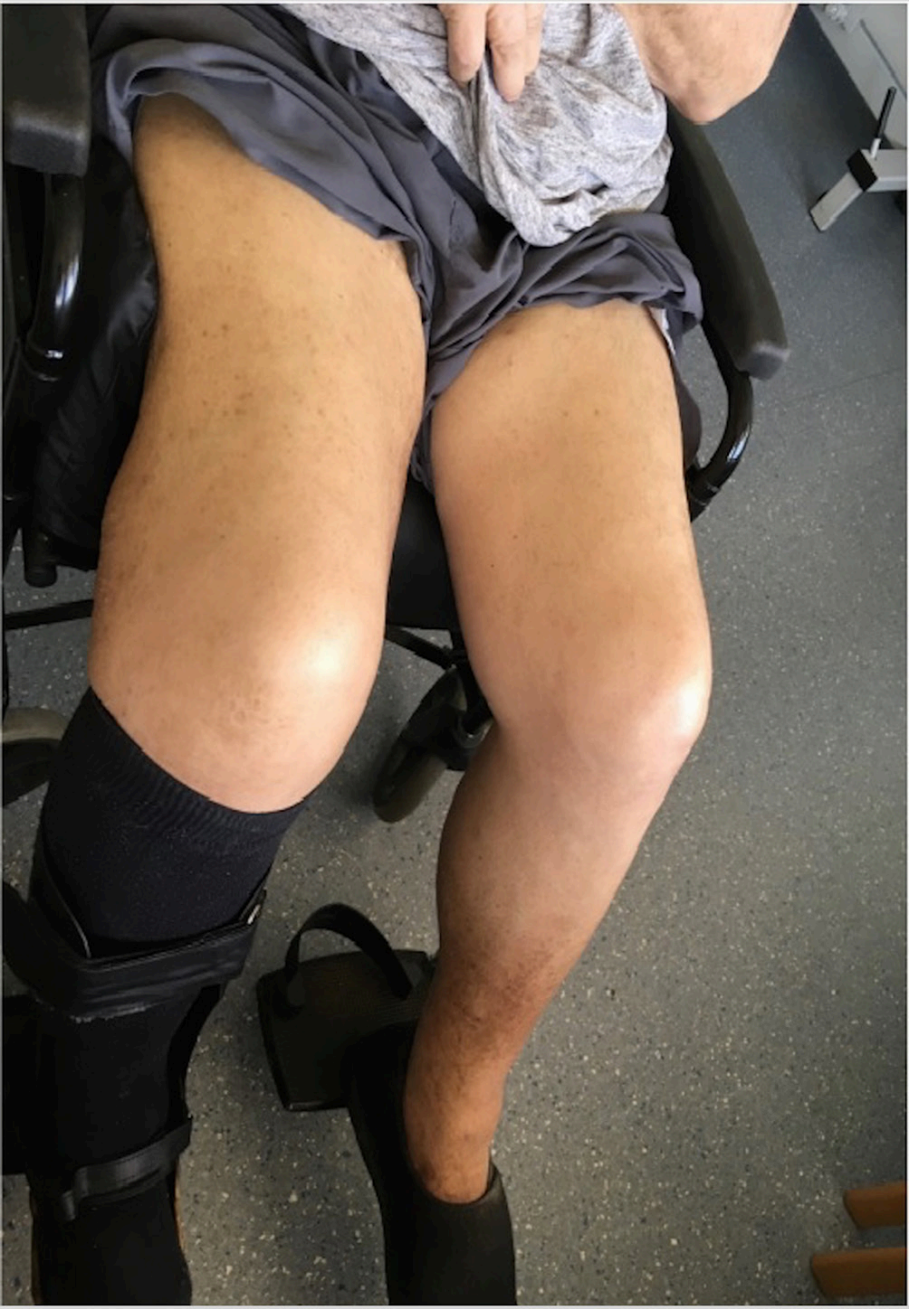
Two months’ post-recanalisation, the patient’s right thigh circumference has decreased dramatically from 68.5 to 52.5 cm (compared to 57.5 cm on the left).

The patient recovered remarkably well following the complex recanalisation. At 2 months’ post-procedure, his right thigh circumference had dramatically reduced and his mobility had improved. His superficial abdominal varicosities clinically resolved, and the skin changes over his right thigh and calf had settled (Figure 6). At 6 months’ he was reported to be doing well and Doppler US demonstrated patency of his venous stents. At 18 months’ his thigh circumference had returned to normal, and he reported to be free from clinical symptoms with vastly improved mobility. He continues to take long-term oral anticoagulation, and there has been no evidence of recurrent thrombosis to date. His AVF remains *in situ*, and may be helping to maintain patency of his venous grafts. He has been seen regularly by the cardiology team to monitor for signs of right heart failure following the sudden increase in venous return, however, he has not shown any signs of cardiac compromise to date.

## Discussion

The PREPIC study, a randomised control trial following 400 patients with proximal DVT, quoted an incidence of IVC thrombosis at 13% 8 years after insertion.^3^ Thrombosis may develop either as a result of extension of the DVT within the distal veins, trapping of peripheral emboli within the filter, or thrombosis *in situ* due to the pro-thrombotic nature of the filter itself.^4^ In the most severe cases, the thrombus can cause complete occlusion of the IVC. This restricts venous return from the lower extremities, causing venous hypertension and stasis in the vessels. PTS may subsequently develop, manifesting as symptoms and signs of chronic venous insufficiency in the lower limbs, including extreme pain, swelling, ulceration and the development of collateral pathways which appear clinically as varicosities in the abdomen.

Complication rates tend to rise in line with the duration of filter implantation, and therefore, NICE guidelines recommend retrieval of the IVC filter as soon as pharmacological anticoagulation is appropriate.^5^ However, in our patient, IVC thrombosis was discovered within a month of insertion, and the presence of thrombus was a contraindication to removing the filter. Once the patient had been appropriately anticoagulated, he should have been reviewed with a view to removing the filter as soon as possible, as this may have prevented the development of his severe PTS 2 years later.

In this case, it was originally hypothesised that the AVF arose as an iatrogenic complication of IVC filter insertion. The femoral vein lies posterior to the superficial femoral artery, which can result in the artery being accidentally penetrated when attempting to gain venous access in the groin, thus forming an abnormal connection between the vessels.^6^ However, post-catheterisation AVFs are extremely rare, with an incidence <1%.^6^ Furthermore, in this patient, imaging demonstrated connecting vessels originating from the common femoral artery, superficial femoral artery and profunda femoris. It is questionable whether such extensive connections would have been created during a simple, otherwise uncomplicated venous puncture for IVC filter insertion.

An alternative explanation is that the AVF may have arisen secondary to the presence of thrombus. The exact mechanism is unclear and very few human studies have explored this phenomenon. Animal studies have demonstrated that an inflammatory reaction occurs post-thrombosis, and a range of inflammatory mediators are recruited at the site of injury.^7–9^ The severity of the reaction is proportional to the extent of thrombus. It has been theorised that this inflammatory reaction causes an increase in the number of small arteries supplying the walls of the vein, and this process of neovascularisation allows the damaged vein to heal and remodel.^10,11^ As the blood flows through the arteries at a higher pressure than the blood behind the thrombus, the arteries become the dominant inflow to the vein as the thrombus recanalises, thus forming an AVF.

The extent to which the AVF is thought to be contributing to this patient’s symptoms is debateable. The presence of an AVF could contribute to venous hypertension through arterialisation of the venous system – a phenomenon that is well documented in relation to surgically-created AVFs in dialysis patients.^12^ The effect of higher arterial pressure on normally low-pressure veins may damage distal valves contributing to venous stasis.

On the other hand, the effect of higher arterial pressures may have been helping to maintain a good (and rather higher than necessary) flow in an already obstructed venous system, and thus would have reduced the possibility of recurrent DVT.

However, Labropoulos et al studied 22 patients with AVF in thrombosed veins and only 4 of these experienced local symptoms. It was observed that there was no obvious impact on the gross arterial system surrounding the AVF, and therefore concluded that the flow in the AVF must be low.^13^ This might suggest that the AVF was an incidental occurrence, rather than a significant contributing factor in this patient’s symptomatology.

Endovascular recanalisation is the preferred treatment for patients with chronically obstructed IVCs. Although we recommend early intervention for IVC occlusion, this particular case highlights that recanalisation can be just as effective even if there is a long interval between the onset of occlusion and the stent being placed. Murphy et al followed 71 patients who underwent IVC recanalisation for symptomatic chronic IVC occlusion for an average of 48 months and suggested that the longer the interval, the more time there is for the development of collateral venous drainage in the lower extremities, which can improve flow across the stent and help to maintain patency.^14^ In addition, in this case we have shown the effectiveness of recanalisation in the context of an arterialised system, and suggest that the AVF may also be helping to maintain patency of the vessels.

## Learning points

1. IVC thrombosis and chronic IVC occlusion are frequently encountered long-term complications of indwelling IVC filters.
2. Patients with IVC filters must be reviewed regularly to ensure that filters are removed as soon as it is clinically appropriate and safe to do so.
3. AVFs are a rare but possible complication of venous thrombosis.
4. Endovascular recanalisation and stenting is a safe and effective treatment option for patients with chronic IVC occlusion and concomitant AVF.
